# A comparative study on short-term therapeutic effects of percutaneous transforaminal endoscopic discectomy and microendoscopic discectomy on lumbar disc herniation

**DOI:** 10.12669/pjms.35.2.650

**Published:** 2019

**Authors:** Fei Wang, Dong Guo, Tiansheng Sun, Kai Guan

**Affiliations:** 1 *Fei Wang, Department of Orthopedics, The Seventh Medical Center of PLA General Hospital, No. 5 Nanmencang, Dongcheng District, Beijing 100700, P. R. China*; 2 *Dong Guo, Department of Orthopedics, The Seventh Medical Center of PLA General Hospital, No. 5 Nanmencang, Dongcheng District, Beijing 100700, P. R. China*; 3 *Tiansheng Sun, Department of Orthopedics, The Seventh Medical Center of PLA General Hospital, No. 5 Nanmencang, Dongcheng District, Beijing 100700, P. R. China*; 4 *Kai Guan, Department of Orthopedics, The Seventh Medical Center of PLA General Hospital, No. 5 Nanmencang, Dongcheng District, Beijing 100700, P. R. China*

**Keywords:** Lumbar disc herniation, Microendoscopic discectomy, Percutaneous transforaminal endoscopic discectomy

## Abstract

**Objective:**

To find out the short-term effects of percutaneous transforaminal endoscopic discectomy (PTED) and microendoscopic discectomy (MED) on lumbar disc herniation (LDH).

**Methods:**

Ninety LDH patients treated in PLA Army General Hospital from July 2015 to July 2016 were selected and randomly divided into an MED group and a PTED group. Length of incision, amount of intraoperative bleeding, surgical time, number of times using intraoperative fluoroscopy, postoperative bedridden time, hospital stay, together with visual analogue scale (VAS) and Oswestry disability index (ODI) scores before surgery, three days, three months and six months after surgery were analyzed.

**Results:**

As regards the length of surgical incision, amount of bleeding, postoperative bedridden time and hospital stay, the PTED group was significantly superior to the MED group ((P=0.000, 0.000, 0.000, 0.001, respectively)). Compared with the PTED group, the MED group used less fluoroscopy and had shorter surgical time (P=0.001, 0.000, respectively). The postoperative VAS and ODI scores of both groups were significantly improved compared with those before surgery (P<0.000, 0.000, respectively). The short-term postoperative low back pain (LBP) VAS score of PTED group was lower than that of MED group (P=0.001). The two groups had similar leg pain (LP) VAS score three and six months after surgery, postoperative and follow-up LP VAS and ODI scores, and surgical improvement rate (P=0.093, 0.097, respectively).

**Conclusion:**

LDH was effectively treated by both PTED and MED. Compared with MED, PTED had less trauma, less blood loss, and faster recovery after surgery.

## INTRODUCTION

Lumbar disc herniation (LDH), also known as rupture of fibrous annulus of lumbar spine or lumbar nucleus pulposus prolapse, is one of the common and frequently occurring diseases of orthopedics,^1^ which is the main reason for low back pain (LBP) and lower extremity radicular symptoms.^2^ About 40% of cases with LB symptoms are caused by LDH.^3^ The lifetime prevalence of LDH is about 1-3%. Most clinically relevant LDH occurs at 30-59 years old, but can also occur in adolescents and the elderly.^4^ About 2-5% of LDH patients need to seek treatment. The methods for LDH treatment include conservative treatment and surgical treatment. In terms of surgical treatment, the minimally invasive technology is often used, which includes microendoscopic discectomy (MED)^5^ and percutaneous transforaminal endoscopic discectomy (PTED).^6^

On this basis, this study made a comparison between the two surgical methods retrospectively. The patients who were diagnosed as single-segment LDH from July 2015 to July 2016 in our hospital were selected, and divided into a PTED group and an MED group. The clinical data were collected, and visual analogue scale (VAS) score and Oswestry disability index (ODI) were analyzed to study the efficacy of the two minimally invasive surgical techniques. Follow-up was made postoperatively to obtain their results of clinical indices, which were compared to provide the basis for clinical treatment.

## METHODS

This study was approved by the ethics committee of our hospital, and written consent has been obtained from all patients. Ninety LDH patients admitted in our hospital from July 2015 to July 2016 were randomly selected as the study subjects, of whom there were 53 males (58.89%) and 37 females (41.11%) with an average age of (48.36 ± 3.21) years old. By using the random number method, the patients were divided into an MED group and a PTED group (n=45). Comprehensive communications were conducted with the patients and their family members preoperatively, who agreed with the surgical procedure and signed informed consent.

### Inclusion criteria

1. 30-65 years old, without gender bias
2. All patients had obvious pain in waist and lower extremities, radiating pain in affected extremities, numbness and discomfort, and some patients had decreased muscle strength or an absent tendon reflex, who received regular conservative treatment preoperatively for at least one month, with poor results
3. All patients were diagnosed as single-segment LDH (L4/L5 or L5/S1) by CT or MRI, and the results of imaging diagnosis were consistent with symptoms
4. No combined lumbar instability, spondylolisthesis; no surgery or intraspinal epidural interventional treatment
5. Removal of percutaneous transforaminal endoscopic pulposus or MED treatment
6. Complete clinical data and access to six months of follow-up after surgery
7. No other surgical contraindications.

### Exclusion criteria

1. The patients who did not meet the inclusion criteria
2. The patients with incomplete imaging information or unable to complete follow-up
3. The X-ray, CT and MRI examinations showed the presence of moderate and severe central spinal stenosis, severe disc calcification, posterior interruption of vertebral centrum or lumbar instability
4. The patients with previous history of lumbar intervertebral disc surgery
5. The patients with significantly abnormal results of laboratory tests or with poor local skin conditions.

### Surgical methods

The surgeries of all subjects were performed by the same surgeon who was skilled at MED and PTED. The detailed surgical methods for MED and PTED groups are shown in Supporting Information.

### Postoperative treatment

The patients of the MED group were treated with antibiotics or corset only once temporarily, had bed rest three to four days after surgery, and needed to wear a waistline in case of walking within one month. During the bedridden period, the patients should do exercises of straight-leg raise and moderate lumbodorsal muscles. Violent activities should be forbidden within 3 months after surgery.

The patients of the PTED group were treated with antibiotics only once temporarily, had bed rest 1 to 2 days after surgery, and needed to wear a waistline or corset in case of walking within 1 month. During the bedridden period, the patients should do exercises of straight-leg raise and moderate lumbodorsal muscles. Violent activities should be forbidden within three months after surgery.

### VAS scoring

VAS consists of a 10 cm-long horizontal line with “painless” and “intolerable pain” marked on both ends. “0” is “painless”, “10” is unbearable pain. The patient should mark the points of pain intensity corresponding to their own feeling on the line. The comprehensive degree of pain was evaluated preoperatively and three days, three months and six months after surgery.

### ODI scoring

ODI is a questionnaire for self-quantification of LBP patients, with a total of nine items, each with six alternative answers (0-5 scores; 0 for no any dysfunction, 5 points for the most obvious dysfunction). The list mainly includes the evaluation of such three aspects as pain (pain degree, the impact of pain on sleep), individual ability (weight lift, sitting, standing, walking) and comprehensive personal ability (ability of daily activities, social activities and excursion), which is more comprehensive than single pain assessment. The total score is 45 points. The actual index of patients is the percentage of their score to the total nine highest scores (45 points). The ODI is the percentage of the accumulated scores of the answers to the nine items to the total 9 highest scores (45 points). 0% is normal. The closer to 100%, the more severe the dysfunction is. In this study, we used the ODI to evaluate the daily life ability of patients preoperatively, three months and six months after surgery.

### Statistical analysis

All data were analyzed by SPSS16.0. The numerical data were expressed as (±SD), and the two-independent sample t-test was used for two-group comparisons. Pairwise comparisons in the same group at different time points were performed by the paired samples t-test. The categorical data were expressed as frequency with percentage, and Chi-square test was used for two-group comparisons. P<0.05 was considered statistically significant.

## RESULTS

### Baseline clinical data

There were 45 patients in the MED group, including 26 males (57.78%) and 19 females (42.22%) with an average age of (47.54 ± 3.29) years old. The average disease course was (19.33 ± 1.86) months. There were 29 cases of L4/L5 LDH and 16 cases of L5/S1 LDH.

There were 45 patients in the PTED group, including 27 males (60.00%) and 18 females (40.00%) with an average age of (48.52 ± 2.65) years old. The average disease course was (19.47 ± 1.82) months. There were 27 cases of L4/L5 LDH and 18 cases of L5/S1 LDH. No significant differences were found in age, gender, course of disease and lesion gap between the two groups (P>0.05).

### Surgical parameters, postoperative bed confinement time and hospital stay

All patients successful surgeries, without intraoperative complications such as injuries of spinal dura mater and nerve root, intervertebral infection, subcutaneous or deep hematoma, and damages of major blood vessels and vital organs. As regards the length of surgical incision, the amount of bleeding, postoperative bedridden time and hospital stay, the PTED group was significantly superior to the MED group (P<0.0001, <0.0001, <0.0001, =0.001, respectively). But compared with the PTED group, the MED group less used fluoroscopy and had shorter surgical time, and the differences were statistically significant (P=0.001, <0.0001, respectively) (Table-I).

**Table-I:** Surgical parameters, postoperative bedridden time and hospital stay (x̄±SD).

Group	Case number (n)	Incision length (mm)	Bleeding amount (mL)	Surgical time (min)	Number of fluoroscopy	Postoperative bedridden time (h)	Postoperative hospital stay (d)
MED	45	18.13±1.79	41.85±7.47	49.01±10.16	2.75±0.27	81.36±13.88	6.68±0.30
PTED	45	6.87±0.73	13.02±2.11	92.63±14.50	23.03±10.24	7.52±0.76	3.01±0.52
*t*	-	39.065	24.903	-16.526	-13.273	35.631	41.09
*P*	-	<0.0001	<0.0001	0.001	<0.0001	<0.0001	0.001

### LBP VAS scores

All patients were effectively followed up. The postoperative LBP VAS scores (three days, three months and six months after surgery) of the two groups were significantly improved compared with those before surgery (P=0.000, 0.000, respectively). Besides, the short-term postoperative LBP VAS score 3 days after surgery of the PTED group was lower, and its LBP was significantly improved compared with the MED group, between which the differences were statistically significant (P=0.001) (Table-II).

**Table-II:** LBP VAS scores (x̄±SD)

Group	Case number (n)	Preoperative score	Score 3 days after surgery	t	P	Improvement rate	Score 3 months after surgery	t	P	Score 6 months after surgery	t	P
MED	45	6.34±0.72	3.61±0.21	29.657	<0.0001	(47.84±3.51)%	1.73±0.40	94.009	<0.0001	1.65±0.49	26.037	<0.0001
PTED	45	6.40±0.83	2.05±0.37	59.448	<0.0001	(69.89±2.21)%	1.84±0.46	81.554	<0.0001	1.36±0.54	112.623	<0.0001
*t*	-	-0.770	24.303			-35.66	-1.201			2.684		
*P*	-	0.443	<0.0001			0.001	0.380			0.523		

### Leg pain (LP) VAS scores

All patients were effectively followed up. The postoperative LP VAS scores (three days, three months and six months after surgery) of the two groups were significantly decreased compared with those before surgery. In addition, the short-term postoperative LP VAS score of the MED group was significantly lower than that of the PTED group (P=0.001). There were no significant differences in LP VAS score 3 and 6 months after surgery (P=0.093, 0.097, respectively) (Table-III).

**Table-III:** LP VAS scores (x̄±SD)

Group	Case number (n)	Preoperative score	Score 3 days after surgery	t	P	Improvement rate	Score 3 months after surgery	t	P	Score 6 months after surgery	t	P
MED	45	7.09±0.92	1.39±0.38	61.029	<0.0001	(79.69±2.80)%	1.24±0.30	58.763	<0.0001	0.98±0.20	57.055	<0.0001
PTED	45	7.21±0.96	2.66±0.28	35.003	<0.0001	(69.63±4.93)%	1.35±0.36	37.978	<0.0001	1.05±0.26	34.044	<0.0001
*t*	-	-0.607	-17.894			11.907	-1.622			-1.451		
*P*	-	0.771	0.001			0.001	0.093			0.097		

### ODI scores

The postoperative ODI scores of the two groups were significantly lower than those before surgery. There were no significant differences in postoperative or follow-up ODI scores between the two groups (P=0.282, 0.692, 0.863, respectively) (Table-IV).

**Table-IV:** ODI scores (x̄±SD) (%)

Group	Case number (n)	Preoperative score	Score 3 months after surgery	t	P	Score 6 months after surgery	t	P
MED	45	(57.17±2.96)%	(19.12±2.55)%	120.085	<0.0001	(16.98±1.89)%	128.958	<0.0001
PTED	45	(58.21±3.48)%	(21.18±2.40)%	80.571	<0.0001	(17.05±1.94)%	179.777	<0.0001
*t*	-	-1.526	-3.943			-0.173		
*P*	-	0.282	0.692			0.864		

## DISCUSSION

LDH is a kind of common and multiple diseases. LDH patients often manifest with clinical symptoms such as LBP, numbness, and decreased muscular strength, seriously affecting the quality of life of patients. Therefore, it is inevitable to choose surgical therapy in the case of ineffective conservative treatment. Fundamentally, surgical treatment is the decompression nerve root and resection of the prominent disc nucleus. However, the traditional open surgery has the disadvantages of relatively large injury, long hospital stay and high cost, resulting in fear of some patients. Thus, MED,^7^ PTED^8^,^9^ and other minimally invasive surgeries have attracted wide attention.^10^

MED is conducted via the posterior translaminar approach, which is easily operable and in line with the habits of most orthopedists. Therefore, it has previously been recognized as one of the best surgical methods for LDH. Although MED can directly remove intervertebral disc tissue protruding or prolapsing in the spinal canal, the surgical procedure is similar to that of open surgery by pulling the nerve root and dural sac. As a result, the risks of nerve root damage and adhesion during MED are the same as those during open surgery.^11^,^12^

As regards PTED, it is a minimally invasive method for treating LDH via the lateral approach. During PTED, the nerve root can be decompressed under direct vision without destroying the integrity of posterior spine structure, also retaining the ligamentum flavum as well as reducing clinical symptoms caused by postoperative bleeding, adhesion and scar formation. Meanwhile, the nucleus pulposus tissue degenerated in the disc can be thermocoagulated to repair the ruptured annulus fibrosus, which thus reduces the recurrence rate, leaves a small surgical incision and promotes recovery.^13^,^14^

Despite a long period of MED’s introduction from abroad, it is very critical to master its indications for hospitals that initially conduct such operations.^15^,^16^ The elderly patients with multi-segmental LDH and stenosis are not suitable to choose MED treatment.^17^ In case of recurrence after surgery, it is generally not recommended to perform MED surgery, because it is difficult to enter the spinal canal, which may easily damage the dural sac and cauda equina.^18^ Although it is only a short period for PTED to be carried out in China, its indications are ever expanding with the increased proficiency of surgeon’s expertize.^19^ The contraindications are lumbar spondylolisthesis, instability; prominent L5/S1 segment; scoliosis; the disc dislocated toward the head side: severe adhesion within the canalis vertebralis. All the patients who underwent PTED surgery showed good results, without the occurrence of complications.^20^ The surgical time was not long (85.6 minutes on average), and the intraoperative blood loss was low. The pain level and muscle strength of the patients were significantly improved compared with before surgery.^21^ The surgery had only a small impact on the disk structure, and could be completed under local anesthesia with a small incision, which could minimize the potential damage that might occur in general anesthesia.^22^,^23^ Compared with MED, PTED has a smaller degree of muscle damage and does not destroy the vertical segment of spinalis; protect small joints, without violations of the ligamentum flavum; can use electric coagulation hemostasis, with less blood loss.^24^

As regards the length of surgical incision, the amount of bleeding, postoperative bedridden time and hospital stay, the PTED group was significantly superior to the MED group. Compared with the PTED group, however, the MED group significantly less used fluoroscopy and had significantly shorter surgical time. The postoperative VAS and ODI scores of the two groups were significantly improved compared with those before surgery. Moreover, the short-term postoperative LBP VAS score of the PTED group was lower, and its LBP was significantly improved compared with the MED group, between which the differences were statistically significant. There were no significant differences in LP VAS score 3 and 6 months after surgery, postoperative and follow-up LP VAS and ODI scores, and surgical improvement rate between the two groups.

## CONCLUSION

PTED and MED are both effective for the treatment of LDH. Compared with MED, PTED has the advantages of less trauma, less blood loss, and faster recovery after surgery.

### Authors’ contributions

**FW & KG** designed this study and prepared this manuscript.

**DG & TS** performed this study and analyzed clinical data.
